# No allergy, but mast cells are involved: MRGPRX2 in chronic inflammatory skin diseases

**DOI:** 10.1111/jdv.20541

**Published:** 2025-02-25

**Authors:** Martin Metz

**Affiliations:** ^1^ Institute of Allergology Charité – Universitätsmedizin Berlin, Corporate Member of Freie Universität Berlin and Humboldt‐Universität Zu Berlin Berlin Germany; ^2^ Fraunhofer Institute for Translational Medicine and Pharmacology ITMP, Immunology and Allergology Berlin Germany

About 10 years ago, the mas‐related G protein‐coupled receptor X2 (MRGPRX2) was reported to be crucial for pseudo‐allergic drug reactions,^1^ suggesting that IgE‐mediated activation of mast cells (MC) may not be the only way how MC can contribute to the pathophysiology of diseases. In fact, both MRGPRX2 and MCs may be important in inflammatory skin diseases without an allergic background. In this issue, Kumar and colleagues summarize what is known about the role of MRGPRX2 in pruritus and skin diseases.^2^

Clinical data on the effects of pharmacological blockade of MRGPRX2 are still lacking, but there is increasing evidence for a potential role of the receptor in various skin diseases. The authors provide several arguments for the important role of MRGPRX2 in chronic spontaneous urticaria, atopic dermatitis, rosacea, psoriasis, chronic pruritus, and chronic prurigo.^2^ Most importantly, an increased expression of MRGPRX2 agonists has been identified in all of these diseases. The majority of these agonists are either neuropeptides, such as substance P and vasoactive intestinal polypeptide, or peptides involved in antimicrobial defence (e.g. cortistatin, β‐defensins and LL‐37).^3^ Many resident skin cells such as keratinocytes, sensory nerves, and macrophages, but also inflammatory cells recruited to sites of chronic skin inflammation can release MRGPRX2 agonists. This may also shed some light on the potential of skin MCs to contribute to non‐allergic skin diseases: Although the receptor has also been detected in some other cell types, by far the highest expression of MRGPRX2 is found on skin MCs.^4^ Since all MRGPRX2 agonists can activate MCs to release proteases, cytokines, and histamine,^5^ the observed increase of MRGPRX2 agonists in various inflammatory skin diseases is likely to contribute to at least some aspects (e.g. pruritus) of the respective skin diseases by activating and degranulating skin MCs. Another argument of Kumar et al. for an important involvement of MRGPRX2 is the observed increased sensitivity to provocation with exogenous and endogenous MRGPRX2 agonists in patients with chronic spontaneous urticaria. This increased sensitivity to MRGPRX2 agonists may be due to the increased number of MRGPRX2 expressing cells (i.e. mast cells) observed in the skin of patients with chronic spontaneous urticaria, but also in many other chronic inflammatory skin diseases.

Several MRGPRX2 antagonists have already been tested in preclinical models in vitro and in humanized mouse models in vivo, and have been shown to effectively block MRGPRX2 agonist‐mediated activation of MCs.^2^ Currently ongoing (NCT06077773, NCT06050928) and further planned clinical trials investigating the efficacy of MRGPRX2 antagonists in chronic spontaneous and chronic inducible urticaria will provide insight into the biological relevance of MRGPRX2 in classic MC‐mediated diseases. Furthermore, as indicated in the review by Kumar et al.,^2^ MRGPRX2 antagonists should be further explored in detail in preclinical and clinical studies in other chronic inflammatory diseases (i.e. atopic dermatitis, chronic prurigo and rosacea) and chronic pruritus, diseases that have previously not been considered to be MC‐dependent.

## CONFLICT OF INTEREST STATEMENT

I have received honoraria as a speaker and/or advisor for: AbbVie, Advanz, ALK‐Abello, Allegria, Almirall, Amgen, Argenx, AstraZeneca, Astria, Attovia, Berlin‐Chemie, Blueprint, Celldex, Celltrion, DeepApple, Escient, Galderma, GSK, Incyte, Jasper, Lilly, Novartis, Pfizer, Pharvaris, Regeneron, Sanofi, Santa Ana Bio, Septerna, Teva, ThirdHarmonicBio, Vifor.

## Data Availability

Data sharing not applicable to this article as no datasets were generated or analysed during the current study.
